# Complete Genome Sequences of *Bordetella pertussis* Vaccine Reference Strains 134 and 10536

**DOI:** 10.1128/genomeA.00979-16

**Published:** 2016-09-15

**Authors:** Michael R. Weigand, Yanhui Peng, Vladimir Loparev, Dhwani Batra, Mark Burroughs, Taccara Johnson, Phalasy Juieng, Lori Rowe, M. Lucia Tondella, Margaret M. Williams

**Affiliations:** Centers for Disease Control and Prevention, Atlanta, Georgia, USA

## Abstract

Vaccine formulations and vaccination programs against whooping cough (pertussis) vary worldwide. Here, we report the complete genome sequences of two divergent *Bordetella pertussis* reference strains used in the production of pertussis vaccines.

## GENOME ANNOUNCEMENT

*Bordetella pertussis* is the primary causative agent of whooping cough (pertussis), a respiratory disease most severe in unvaccinated infants. The introduction of vaccines against pertussis dramatically reduced disease incidence worldwide. However, many countries have recently experienced disease resurgence, in part due to genetic divergence of circulating strains. The resulting antigenic mismatch with vaccine references has led many to conclude that *B. pertussis* is evolving under vaccine-driven selection (1–5). Adaptation of *B. pertussis* is complicated by the varied administration of whole-cell and acellular vaccines between countries and the diversity of reference strains used for vaccine production (6–8). Here, we report the complete genome sequences of two such strains used in manufacturing pertussis vaccines: B202 (Lederle Laboratories, strain 134) and B203 (Sanofi-Pasteur MSD, strain 10536) (9).

Whole-genome shotgun sequencing was performed using a combination of the PacBio RSII (Pacific Biosciences, Menlo Park, CA), Illumina HiSeq/MiSeq (Illumina, San Diego, CA), and Argus (OpGen, Gaithersburg, MD) platforms, as described previously (10). Briefly, genomic DNA libraries were prepared for PacBio sequencing using the SMRTbell template prep kit 1.0 and polymerase binding kit P4, while Illumina libraries were prepared using the NEBNext Ultra library prep kit (New England BioLabs, Ipswich, MA). *De novo* genome assembly of filtered reads was performed using the Hierarchical Genome Assembly Process (HGAP version 3; Pacific Biosciences) at 130× and 144× coverage for B202 and B203, respectively. The resulting consensus sequences were determined with Quiver (version 1), manually checked for circularity, and then reordered to match the start of reference strain Tohama I (accession no. CP010964) (10). To ensure accuracy, assemblies were confirmed by comparison to BamHI and KpnI restriction digestion optical maps using the Argus system (OpGen) with MapSolver (version 2.1.1; OpGen) and further polished by mapping either Illumina HiSeq PE-100 or MiSeq PE-300 reads using CLC Genomics Workbench (version 8.5; CLC bio, Boston, MA). Final assemblies were annotated using the NCBI automated Prokaryotic Genome Annotation Pipeline (PGAP).

The average G+C content of both B202 and B203 was 67.1%, with genome sizes of 4,128,979 and 4,134,643 bp, respectively. Genome annotation identified 3,645 protein-coding genes in B202 and 3,636 protein-coding genes in B203. Both genomes encoded three rRNA operons and 51 tRNAs.

The assemblies were distinct from genomes of vaccine reference strains Tohama I (GlaxoSmithKline, accession no. CP010964), CS (China, accession no. CP010963), and 137 (Brazil, accession no. CP010323), which have been sequenced previously (10, 11). B202 and B203 were not related, and their genomes differed from that of Tohama I by multiple rearrangements, as well as 186 and 410 single-nucleotide polymorphisms (SNPs), respectively. The genome of B202 was phylogenetically and structurally similar, but not identical, to other strains with the profile *prn1-ptxP1-ptxA2-ptxB2-fimH1*, such as clinical isolate H375 (accession no. CP010961) (10). B203 appeared to be closely related to Brazilian vaccine strain 137, sharing allele profile *prn7-ptxP2-ptxA4-ptxB2-fimH1*, but differed by 13 SNPs and a single ~74-kb inversion flanked by rRNA operon copies.

The availability of these genome sequences provides added resolution to known diversity among references used in vaccine production and will hopefully aid in the research of immune response to clinical infection in vaccinated patients.

### Accession number(s).

The complete genome sequences have been deposited at DDBJ/EMBL/GenBank under the accession numbers CP016338 and CP012128 for *B. pertussis* B202 and B203, respectively. The versions described in this paper are the first versions.

## References

[1] Bart MJ, Harris SR, Advani A, Arakawa Y, Bottero D, Bouchez V, Cassiday PK, Chiang CS, Dalby T, Fry NK, Gaillard ME, van Gent M, Guiso N, Hallander HO, Harvill ET, He Q, van der Heide HG, Heuvelman K, Hozbor DF, Kamachi K, Karataev GI, Lan R, Lutynska A, Maharjan RP, Mertsola J, Miyamura T, Octavia S, Preston A, Quail MA, Sintchenko V, Stefanelli P, Tondella ML, Tsang RS, Xu Y, Yao SM, Zhang S, Parkhill J, Mooi FR 2014 Global population structure and evolution of *Bordetella pertussis* and their relationship with vaccination. mBio 5:e01074-14. doi:10.1128/mBio.01074-14. 24757216 PMC3994516

[2] Octavia S, Maharjan RP, Sintchenko V, Stevenson G, Reeves PR, Gilbert GL, Lan R 2011 Insight into evolution of *Bordetella pertussis* from comparative genomic analysis: evidence of vaccine-driven selection. Mol Biol Evol 28:707–715. doi:10.1093/molbev/msq245. 20833694

[3] Sealey KL, Harris SR, Fry NK, Hurst LD, Gorringe AR, Parkhill J, Preston A 2015 Genomic analysis of isolates from the United Kingdom 2012 pertussis outbreak reveals that vaccine antigen genes are unusually fast evolving. J Infect Dis 212:294–301. doi:10.1093/infdis/jiu665. 25489002

[4] van Gent M, Bart MJ, van der Heide HG, Heuvelman KJ, Mooi FR 2012 Small mutations in *Bordetella pertussis* are associated with selective sweeps. PLoS One 7:e46407. doi:10.1371/journal.pone.0046407. 23029513 PMC3460923

[5] Xu Y, Liu B, Grondahl-Yli-Hannuksila K, Tan Y, Feng L, Kallonen T, Wang L, Peng D, He Q, Wang L, Zhang S. 2015 Whole-genome sequencing reveals the effect of vaccination on the evolution of *Bordetella pertussis*. Sci Rep 5:12888. 26283022 10.1038/srep12888 PMC4539551

[6] Van Amersfoorth SC, Schouls LM, van der Heide HG, Advani A, Hallander HO, Bondeson K, von König CH, Riffelmann M, Vahrenholz C, Guiso N, Caro V, Njamkepo E, He Q, Mertsola J, Mooi FR 2005 Analysis of *Bordetella pertussis* populations in European countries with different vaccination policies. J Clin Microbiol 43:2837–2843. doi:10.1128/JCM.43.6.2837-2843.2005. 15956406 PMC1151907

[7] Kallonen T, Gröndahl-Yli-Hannuksela K, Elomaa A, Lutyńska A, Fry NK, Mertsola J, He Q 2011 Differences in the genomic content of *Bordetella pertussis* isolates before and after introduction of pertussis vaccines in four European countries. Infect Genet Evol 11:2034–2042. doi:10.1016/j.meegid.2011.09.012. 21964035

[8] Kilgore PE, Salim AM, Zervos MJ, Schmitt HJ 2016 Pertussis: microbiology, disease, treatment, and prevention. Clin Microbiol Rev 29:449–486. doi:10.1128/CMR.00083-15. 27029594 PMC4861987

[9] Litt DJ, Neal SE, Fry NK 2009 Changes in genetic diversity of the *Bordetella pertussis* population in the United Kingdom between 1920 and 2006 reflect vaccination coverage and emergence of a single dominant clonal type. J Clin Microbiol 47:680–688. doi:10.1128/JCM.01838-08. 19158267 PMC2650949

[10] Bowden KE, Weigand MR, Peng Y, Cassiday PK, Sammons S, Knipe K, Rowe LA, Loparev V, Sheth M, Weening K, Tondella ML, Williams MM 2016 Genome structural diversity among 31 *Bordetella pertussis* isolates from two recent U.S. whooping cough statewide epidemics. mSphere 1:e00036-16. doi:10.1128/mSphere.00036-16. 27303739 PMC4888882

[11] Akamatsu MA, Nishiyama MY Jr, Morone M, Oliveira UC, Bezerra MF, Sakauchi MA, Raw I, Junqueira de Azevedo IL, Kitajima JP, Carvalho E, Ho PL 2015 Whole-genome sequence of a *Bordetella pertussis* Brazilian vaccine strain. Genome Announc 3(1):e01570-14. doi:10.1128/genomeA.01570-14. 25700409 PMC4335333

